# The construction and evaluation of secretory expression engineering bacteria for the trans-Cry3Aa-T-HasA fusion protein against the *Monochamus alternatus* vector

**DOI:** 10.3389/fcimb.2024.1362961

**Published:** 2024-02-23

**Authors:** Xiaohong Han, Chenyan Huang, Huan Qi, Yukun Zhu, Xinran Hu, Yingxin Wen, Yirong Long, Lei Xu, Feiping Zhang

**Affiliations:** ^1^ College of Forestry, Fujian Agriculture and Forestry University, Fuzhou, China; ^2^ Key Laboratory of Integrated Pest Management in Ecological Forests, Fujian Province University, Fujian Agriculture and Forestry University, Fuzhou, China; ^3^ State Key Laboratory of Biocontrol, School of Ecology, Sun Yat-sen University, Guangzhou, China; ^4^ Graduate School of Chinese Academy of Agricultural Sciences, Beijing, China

**Keywords:** *Monochamus alternatus*, gut microbiota, engineer bacteria, Yersinia entomophaga, pine wilt disease

## Abstract

Pine wood nematode disease is currently the most deadly forest disease in China, and the *Monochamus alternatus* is its primary vector. Controlling the *M. alternatus* is crucial for managing pine wood nematode disease. This study, based on the selected HasA (pGHKW4) secretory expression vector, used electroporation to combine the genetically modified high-toxicity toxin Cry3Aa-T with the entomopathogenic bacterium *Yersinia entomophaga* isolated from the gut of the *M. alternatus*. The SDS-PAGE and Western blotting techniques were employed to confirm the toxin protein’s secretion capability. The engineered bacteria’s genetic stability and effectiveness in controlling *M. alternatus* were assessed for their insecticidal activity. The results of the SDS-PAGE and Western blotting analyses indicate that the HasA system effectively expresses toxin protein secretion, demonstrates certain genetic stability, and exhibits high insecticidal activity against *M. alternatus.* This study constructed a highly toxic entomopathogenic engineered bacterial strain against *M. alternatus* larvae, which holds significant implications for controlling *M. alternatus*, laying the foundation for subsequent research and application of this strain.

## 1 Introduction

Pine wilt disease, caused by pine wood nematodes, is an immensely destructive forest disease that originated in North America and has since spread to several regions, such as China, Japan, Korea, and Europe (Li et al., 2021). With the ongoing rapid expansion of the affected area, the disease has led to the demise of over 100 million pine trees in China, making it one of the most deadly forest diseases in the country. Consequently, this disease significantly threatens China’s ecological environment (Li et al., 2021). Pine wood nematodes are transmitted by several beetle species belonging to the *Monochamus* genus. In China, the primary insect vector for the spread of pine wilt disease is *Monochamus alternatus*. Accordingly, controlling the vector insect, *M. alternatus*, is a critical approach to preventing and managing pine wilt disease (Zhang et al., 2022).

For controlling *M. alternatus*, various methods are available, including efficient trapping, chemical control, and biological control (Liu and Shu, 2007; Chen and Peng, 2014). Among these, using pathogenic microorganisms to control pests presents advantages, such as high safety, strong selectivity, and environmental friendliness, and has been widely utilized. In our initial research, we successfully isolated a strain of entomopathogenic *Yersinia entomophaga* from the gut of *M. alternatus*. This bacterium, belonging to the Enterobacteriaceae family under the *Yersinia* genus, demonstrates efficacy in pest control (Hurst et al., 2020; Schoof et al., 2022, 2023). The bacterium can colonize the gut of *M. alternatus*, exhibiting a certain degree of control over the pest. However, its efficacy in pest management requires further enhancement. Pest control efficacy can be significantly enhanced by genetically modifying symbiotic bacteria in insects to express specific toxins. For example, Watanabe et al. transferred the ice nucleation gene (inaA) into bacteria such as *Enterobacter cloacae*. After ingesting the engineered strain, the cold resistance of *Glyphodes duplicalis* significantly decreased, resulting in a large number of deaths (Watanabe and Sato, 1999; Watanabe et al., 2000). Additionally, Castrillo et al. transferred this gene into *Pseudomonas* spp., leading to a marked reduction in the overwintering survival rate of *Leptinotarsa decemlineata* (Castrillo et al., 2000). Furthermore, Beard et al. demonstrated the capability of symbiotic bacteria, engineered with exogenous anti-parasitic genes, to inhibit parasite growth and transmission, effectively reducing the risk of Chagas disease transmission (Beard et al., 2001).

However, numerous challenges must still be addressed in the current research on utilizing insect gut microbiota for controlling *M. alternatus* (He et al., 2008). The lack of appropriate secretion expression components within the bacterial cells results in the retention of expressed proteins within these cells, preventing their release into the external environment and subsequent action within the insect gut (Wang et al., 2008). This is a primary issue that must be addressed in utilizing this technology to control *M. alternatus*. The alpha-hemolysin (HlyA) secretion system and the HasA-type heme transport system are common Type I secretion systems (T1SS) found in bacteria (Chang et al., 2014; Yu et al., 2015). Bisi et al. discovered that the anti-Pbs21 single-chain variable fragment (scFv) and PLA2 could be successfully expressed in the mosquito gut symbiotic bacterium *Pantoea agglomerans* via the HlyA secretion system (Bisi and Lampe, 2011). Dehghan et al. discovered that *E. cloacae* can express various effector proteins in the mosquito gut via the HasA system (Dehghan et al., 2022). As a result, we utilized green fluorescent protein as a marker protein and constructed engineered strains of entomopathogenic *Yersinia* CSLH88 containing both the HlyA secretion system and the HasA heme transport systems. We successfully identified the more adaptable pGHKW4 plasmid through this process (Huang et al., 2019). Combining this plasmid with the specific toxin *Cry3Aa-T* (Guo et al., 2020), which targets *M. alternatus* larvae, may potentially enhance the efficacy of entomopathogenic *Y. entomophaga* in controlling *M. alternatus*.

Based on the aforementioned ideas, this study utilized the *Y. entomophaga* strain CSLH88 selected by the research group and the *Cry3Aa-T* toxin protein gene, in which the *GFP* gene segment in the pGHKW4 secretory expression vector was replaced with the *Cry3Aa-T* toxin protein gene, leading to constructing of the pCHKW recombinant plasmid. Subsequently, the engineered bacteria’s extracellular protein secretion level, genetic stability, and insecticidal activity of the *Cry3Aa-T* toxin protein were examined, thereby creating a *Y. entomophaga* engineered bacterium (CSLH88-pCHKW) capable of secreting and expressing the *Cry3Aa-T* toxin protein with high toxicity towards *M. alternatus* larvae.

## 2 Materials and methods

### 2.1 The strains and experimental materials

The laboratory-reared second-instar larvae of *the M. alternatus* Hope FAFU strain were employed in this experiment. Both the pLB-*Cry3Aa-T* strain and *Y. entomophaga* CSLH88 were maintained in our laboratory. The pGHKW4 plasmid was previously constructed in our research facility (Huang et al., 2019). We obtained the *Escherichia coli* DH5α competent cells from Nanjing Nuoweizan Co., Ltd. (Catalog number: C502-02). Fuzhou Baijing Biotechnology Co., Ltd synthesized the primers utilized in the experiment. A 100 mg/mL Kanamycin antibiotic (Kan) concentration was employed. After undergoing bacterial filtration, it was incorporated into the solid culture medium at varying proportions.

A 1 L batch of liquid LB medium was prepared by dissolving 10 g of tryptone, 5 g of yeast extract, and 10 g of sodium chloride in distilled water. The resulting mixture was then adjusted to a pH of 7.2 and transferred into conical flasks. After sealing the flasks with a membrane, the medium was sterilized at 121°C for 20 mins, rendering it ready for use.

A solid LB medium (1 L) was prepared using the same proportions as the liquid LB medium previously mentioned. The mixture was poured into conical flasks, and 1.5% agar powder was added. The flasks were then sealed with a membrane, and the medium was sterilized at 121°C for 20 mins to ensure its readiness for use.

To prepare the Kanamycin-containing agar plates for culture, the solid culture medium was sterilized at a high temperature. Once it cooled to 45°C, 100 μL of Kanamycin with a 100 mg/mL concentration was added to the medium. This achieved a final antibiotic concentration of 100 μg/mL. The medium was thoroughly mixed to ensure an even distribution of the antibiotic. Then, pour the mixture was poured into Petri dishes and left to allow it to solidify. The resulting Kanamycin-containing agar plates were prepared for use.

### 2.2 Construction of HasA secretory expression vector based on Cry3Aa-T toxin

In this study, we have designed amplification primers that specifically target the *Cry3Aa-T* gene sequence, which is known to possess insecticidal activity against the II-instar larvae of *M. alternatus*. These larvae have been preserved in the laboratory, specifically in the pLB-*Cry3Aa-T* strain (Guo et al., 2020).

5′- GCTAGCatgaatccgaacaatcgaagtg-3′,

3′-CCCGGGattcactggaataaattcaatt -5′.

The PCR reaction system comprises a total volume of 25 μL. It includes 2 μL of template DNA, 1.25 μL of each primer at a concentration of 10 mmol/L, 12.5 μL of Q5^®^ Hot Start High-Fidelity 2×Master Mix, and 8 μL of ddH_2_O. These reagents should be added sequentially to the PCR tube. The reaction is thoroughly mixed and placed in the PCR machine for amplification. The amplification program consists of 30 cycles, with each cycle comprising the following steps: initial denaturation at 98°C for 5 mins, denaturation at 98°C for 1 min, annealing at 61.8°C for 30 s, and extension at 72°C for 1 min. After 30 cycles, a final extension step should be performed at 72°C for 10 mins. Once the program is complete, the reaction mixture can be stored at 4°C. The pGHKW4 expression vector will be cleaved using the restriction endonucleases *BmtI* and *XmaI*, which will produce two fragments: the *GFP* gene fragment and the vector fragment. After gel purification, the PCR product and the linearized vector DNA fragment will be joined using T4 DNA ligase (NEB). Refer to the instructions in the reagent kit for detailed steps. The transformation and plasmid extraction procedures will follow the protocols detailed in our research group’s previous work [18]. The construction of the HlyA and HasA secretory expressions utilizing Kan resistance will be followed. Subsequently, the obtained plasmid will undergo double digestion with the *BmtI* and *XmaI* restriction enzymes. The plasmid will be subjected to sequencing to verify its sequence. The *GFP* gene fragment in pGHKW4 will be replaced with the *Cry3Aa-T* toxin gene to obtain the pCHKW external secretion expression plasmid.

### 2.3 Transformation and observation of pCHKW secretory expression vector

#### 2.3.1 Preparation of electroporation sensitization state

The CSLH88 strainwas transferred, activated via transfection, into a fresh LB culture medium. The mixture was incubated at 30°C with continuous shaking at 150 r/min for 2-3 h. Subsequently, the bacterial solution was removed and transferred to an ice bath for 10 min. Then, centrifugation was performed at 4°C and 5000 r/min for 5 min to remove the supernatant. Then, the bacterial cells were resuspended in a solution containing 10% glycerol. The centrifugation step was repeated at 4°C and 5000 r/min for 5 min, discarding the supernatant after each repetition. This process was carried out three times. Finally, the bacterial cells were gently resuspended in 100 μL of a 10% glycerol solution to acquire CSLH88 electrotrans formation sensitized cells. These freshly prepared sensitized cells can be stored at -80°C for future use in electroporation experiments.

#### 2.3.2 Electrotransformation and transformant verification

The CSLH88 electrotransformation-sensitized cells were placed on ice and thawed. Next, 5 μL of the recombinant plasmid was added to the cells and carefully mixed using a pipette. The resulting mixture were then swiftly transferred to a chilled electroporation cuvette, ensuring that any water droplets on the outer wall of the cuvette were gently removed using a lens wipe. The following values were adjusted for electroporation: voltage to 2500 V, capacitance to 25 μF, and resistance to 600 Ω. Once the electroporation was completed, 500 μL of LB culture medium were promptly added to the electroporation cuvette. The entire liquid was transferred to a 1.5 mL microcentrifuge tube and incubated at 30°C with shaking at 150 rotations per minute (r/min) for 1.5 h. Subsequently, the reanimated cells were centrifuged at 12000 r/min at room temperature for 1 min; then, the supernatant was discarded. Lastly, 100 μL of the combined cell pellet was retrieved and evenly dispersed onto an LB agar plate containing Kanamycin at a final 100 μg/mL concentration. The plate was inverted and incubated in a constant-temperature incubator at 30°C for 12 h.

A single colony was chosen and inoculated into an LB liquid medium supplemented with Kanamycin (at a final concentration of 100 μg/mL). The culture was then incubated at 37°C with shaking at 150 r/min for 12 h. Plasmid extraction was subsequently performed, followed by selecting appropriate restriction endonucleases for double-digestion. Finally, the sequence was validated using sequencing techniques.

### 2.4 The secretion expression status of the Cry3Aa-T-HasA fusion protein

#### 2.4.1 Extracellular protein concentration

The supernatant was collected by centrifuging the cultured bacterial solution at 4°C and 8000 r/min for 10 mins. Subsequently, the supernatant was carefully transferred to a pre-chilled ultrafiltration tube (Amicon Ultra-4 centrifugal filter devices) and centrifuged at 4°C and 5000 r/min for 20 mins. This process was repeated until the entire supernatant had been concentrated. Once centrifugation was complete, the protein solution in the ultrafiltration tube was gently mixed using a pipette and then transferred to a centrifuge tube. This resulting solution represented the extracellular protein, which should be stored at -20°C for future use.

#### 2.4.2 Extraction of intracellular proteins

After suspending the centrifuged bacterial cells in the PBS buffer, the supernatant should be removed by centrifugation at 4°C and 8000 r/min for 8 mins. This step should be repeated three times. Subsequently, the samples should be placed on ice for sonication treatment at a frequency of 50 Hz, with a cycle of 10 s on and 5 s off. Each bacterial sample should undergo approximately 30 mins of sonication. Once sonication is complete, the cell lysate should be centrifuged at 4°C and 8000 r/min for 8 min. The resulting supernatant contains intracellular proteins and should be stored at -20°C for future use.

#### 2.4.3 SDS-PAGE analysis

To begin the experiment, the SDS-PAGE gel should be prepared, and the protein samples should be loaded onto it. For SDS-PAGE analysis, a voltage of 90 V should be applied. Subsequently, the gel should be stained with Coomassie Brilliant Blue R250 dye and destained by gently shaking it in a destaining solution. Lastly, the SDS-PAGE results can be analyzed using a bidirectional electrophoresis scanning imaging system.

#### 2.4.4 Western blot analysis

Western blot analysis was conducted simultaneously. We began this process by performing SDS-PAGE as the standard and subsequently transferred the separated proteins from the SDS-PAGE gel to a nitrocellulose (NC) membrane using a current of 200 mA for 1 h. Then, the standard protocol was followed, including blocking with 5% milk and incubating with primary and secondary antibodies. In this instance, the primary antibody solution was substituted with a diluted *Cry3Aa-T*ag Rabbit Polyclonal Antibody solution at a dilution of 1:2000 while keeping the remaining steps and reagents unchanged.

### 2.5 Genetic stability testing of toxin-engineered bacterial strains

In this study, the CSLH88-pCHKW bacterial strain was inoculated into a liquid LB medium without antibiotics, using 1% inoculum, after being activated for 12 hours. The culture was then incubated on a shaker at 150 rpm and 30°C for 12 h. Subsequently, the subculturing process was repeated. After each odd-numbered subculturing (e.g., 1st, 3rd, 5th, etc.), the bacterial suspension was taken and subjected to serial dilution. The diluted samples were then plated on a solid LB medium without antibiotics. This dilution plating process was repeated three times for each subculturing process. The plates were incubated in a temperature-controlled incubator at 30°C for 24 h. A total of 100 distinct colonies were selected and individually inoculated onto solid culture media supplemented with Kanamycin (to facilitate selection) and onto solid culture media without any antibiotics. After incubation for 12 h, we ascertained the number of bacterial colonies on each plate. The gene carriage percentage was calculated by dividing the number of bacterial colonies observed on the Kanamycin-containing solid culture medium by the number of bacterial colonies observed on the solid culture medium without antibiotics. This computation will evaluate the recombinant plasmid’s genetic stability in the absence of selective pressure. The CSLH88 strain was employed and subjected to identical conditions as a negative control.

### 2.6 Biological assessment of engineered strains for toxin production

The engineered and non-engineered strains were cultured separately in 100 mL conical flasks for 24 h. The bacterial solution OD_600_ was determined and diluted. Then, 100 μL of the diluted solution was applied to LB agar for cultivation. Subsequently, the original bacterial concentration was calculated. Sterile LB was used blank controls. This process was repeated three times. The second-instar *M. alternatus* larvae were fed. 300 μL of bacterial suspension with varying concentrations was dispensed onto 1 g of tissue paper to ensure even distribution. The tissue paper was then transferred into a 1.5 mL centrifuge tube. Subsequently, second-instar *M. alternatus* larvae were carefully placed into the centrifuge tube. The specimens were cultivated under the following conditions: a temperature of 25 °C, a humidity of 75%, and a light–dark cycle of 16:8 h. Unmodified bacterial strains were employed as blank controls subjected to the same treatment. Throughout the rearing process, the toxic effects of the bacterial strains on *M. alternatus* larvae were observed, and their mortality rates were subsequently recorded. Each treatment with 10 larvae was repeated three times.

### 2.7 Detection of the proliferation of the CSLH88-pCHKW strain in the gut of *M. alternatus*

The CSLH88-pCHKW strain was taken from an overnight culture, and after cultivation, the concentration of the CSLH88-pCHKW strain was diluted to 10^5^. The diluted bacterial solution was uniformly mixed into the diet of *M. alternatus* larvae, which were subsequently fed. Afterward, the larvae’s intestines were dissected at different time intervals. The dissected intestines were ground and spread onto kanamycin agar plates containing 100 μg/mL. After incubating the agar plates in a culture chamber for 24 h, individual colonies were picked and subjected to PCR verification. The specific amplification primer for *Cry3Aa-T* was used for validation, and the number of bacterial colonies containing *Cry3Aa-T* was counted to determine their proliferation in the gut of *M. alternatus*.

### 2.8 Statistical analysis

The data from this experimental study were measured in multiple parallel replications. Statistical analysis was performed using SPSS 22.0 software, employing one-way analysis of variance (ANOVA), with P<0.05 indicating statistical significance. LC50 was calculated utilizing the probit regression model in the SPSS software. Graphs were generated using the Graphpad Prism 8 software.

## 3 Results

### 3.1 Construct a recombinant vector for the secretion of the HasA protein based on the Cry3Aa-T toxin

**Figure 1:**
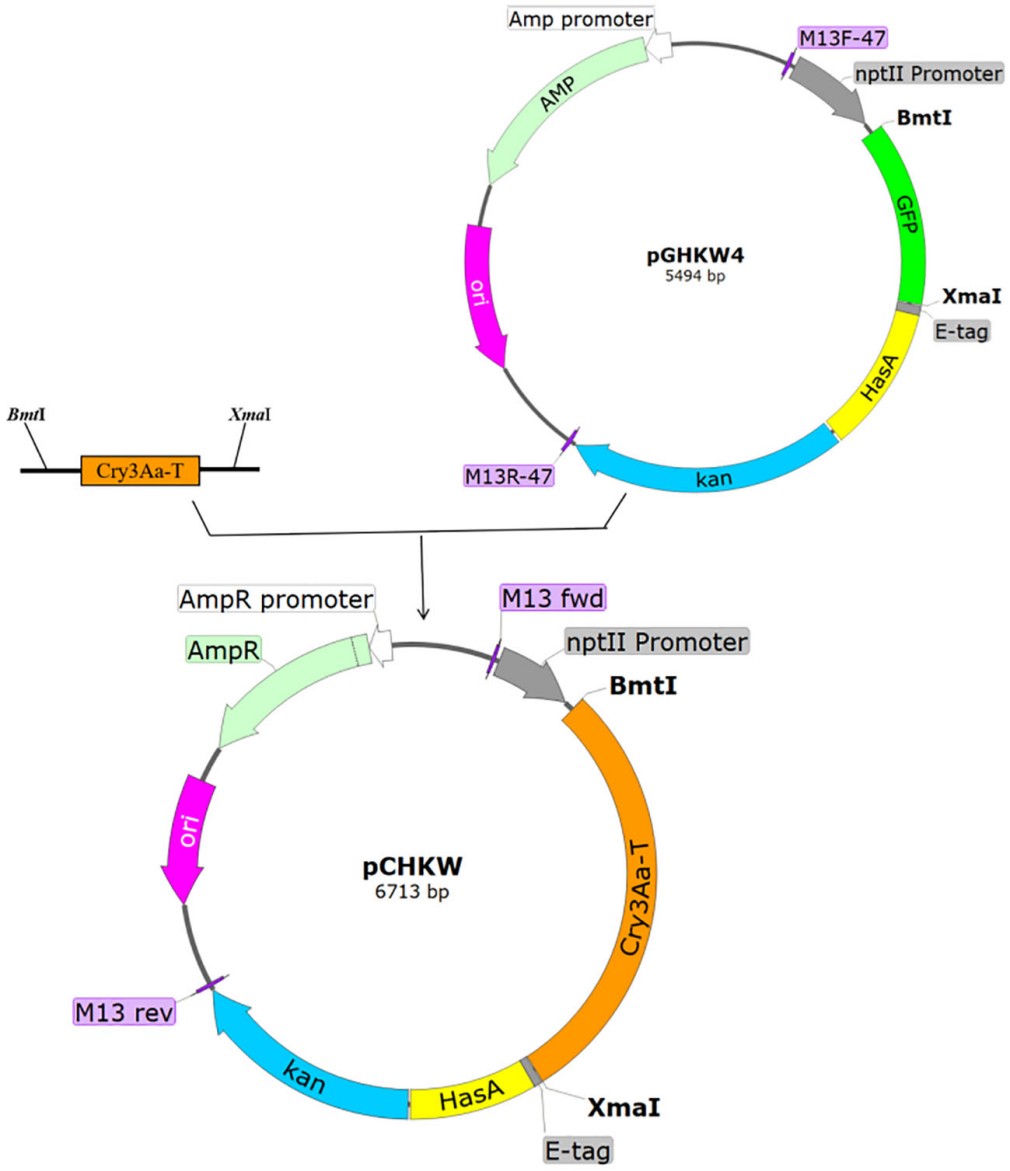
pCHKW plasmid construction process. Using the pGHKW4 plasmid, the GFP gene was replaced with the Cry3Aa-T gene to construct the pCHKW plasmid. GFP, green fluorescent protein; Cry3Aa-T, insecticidal toxin protein; E-tag, protein tag; HasA, secretory expression element; Kan, kanamycin resistance gene; BmtI/XmaI, restriction endonucleases; MCS, multiple cloning site; M13 fwd/M13 rev (M13F-47/M13R-47), universal forward and reverse primers for the pUC19 vector; ori, origin of replication; AmpR, ampicillin resistance gene; AmpR promoter, ampicillin resistance gene promoter; nptII promoter, nptII promoter.

The *Cry3Aa-T* toxin protein gene was amplified using PCR. Subsequently, the *GFP* gene fragment in the pGHKW4 secretion expression vector was replaced with the *Cry3Aa-T* toxin protein gene. This replacement was achieved by employing *BmtI*, *XmaI* restriction endonucleases, and T4 DNA ligase. The process led to the creating of the pCHKW recombinant plasmid creation, as depicted in **Figure 1**. The pCHKW plasmid, derived from the pUC19 vector backbone, features a nptII promoter, the *Cry3Aa-T* gene, a *kan* resistance gene, and the constituents of the HasA secretion system. The size of the plasmid is 6713 bp.

**Figure 2:**
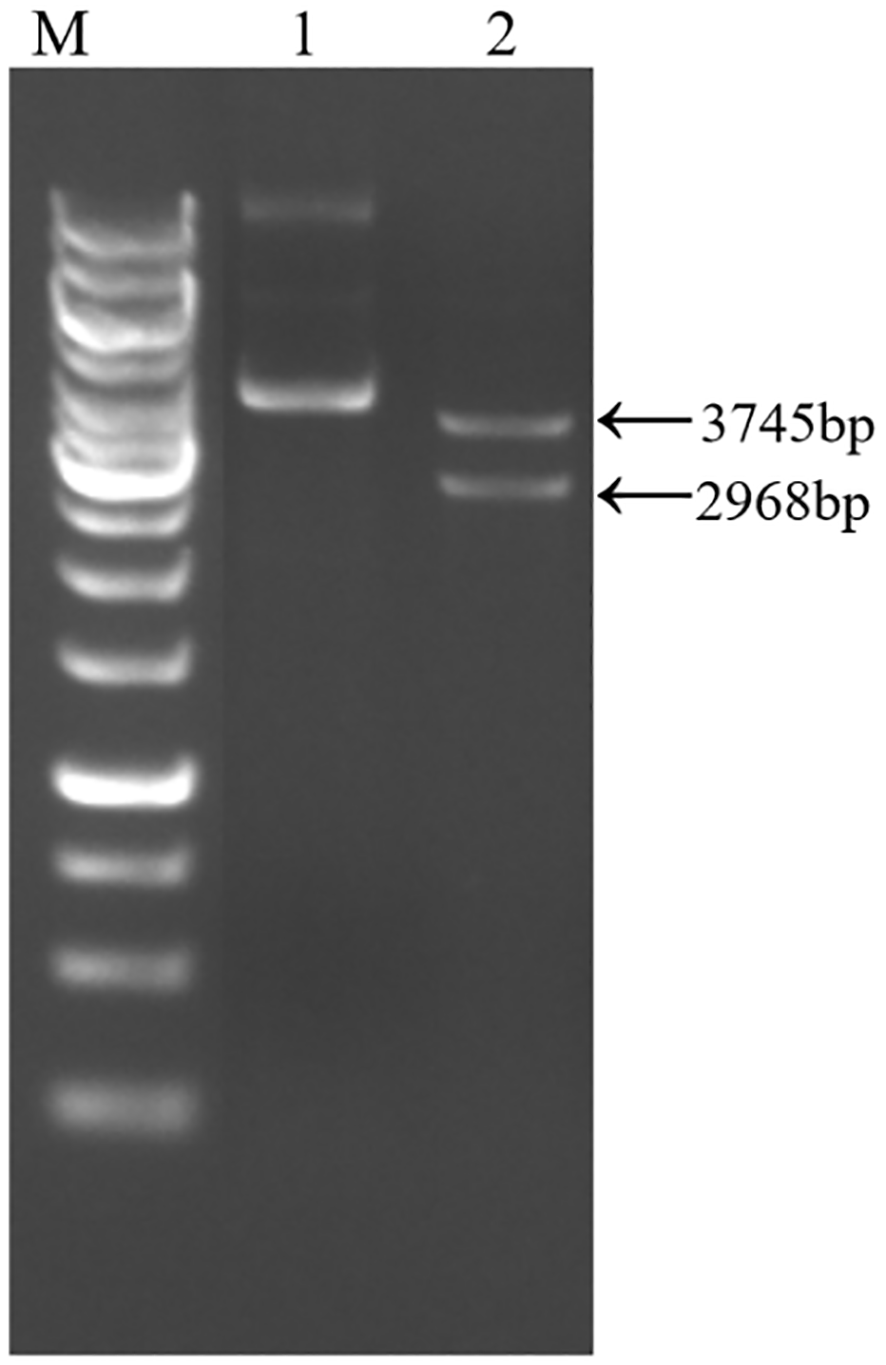
pCHKW plasmid profile and electrophoresis. M: 1kb DNA marker. 1: pCHKW plasmid. 2: pCHKW plasmid digestion products (SacI and SpeI).

Following the electrotransformation of the plasmid into *Y. entomophaga*, the constructed positive clone plasmid was validated through double digestion with SacI and SpeI restriction enzymes. The anticipated fragment sizes of 2968 bp and 3745 bp align with the band sizes observed in agarose gel electrophoresis (**Figure 2**). The sequencing validation results establish the congruity between the obtained sequence and the designed band sequence, thus providing evidence of the successful construction of the pCHKW secretion expression vector.

### 3.2 Comparative analysis of the secretion and expression patterns of Cry3Aa-T toxin in *Y. entomophaga* strains

**Figure 3:**
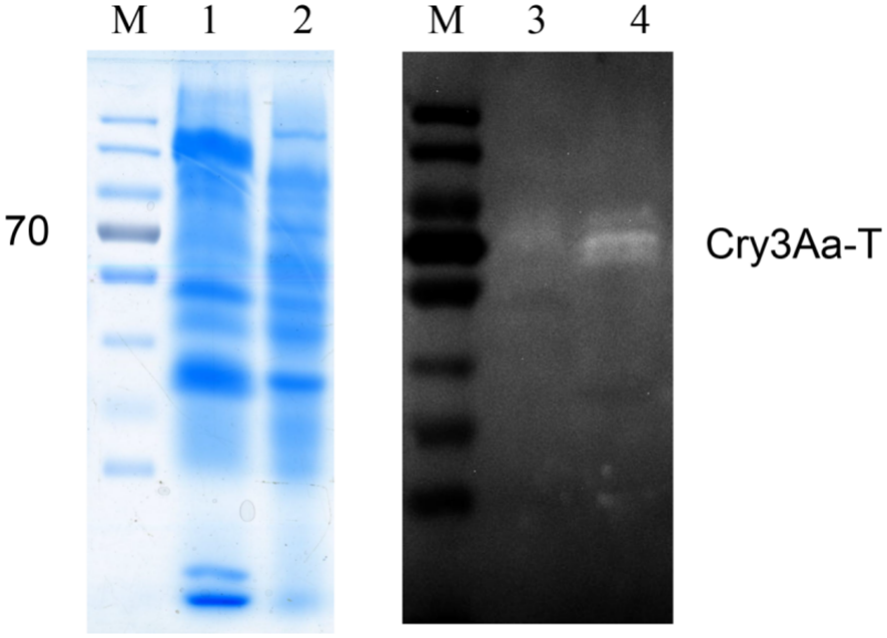
SDS-PAGE and Western blot electrophoresis images of the engineered strains. M: Protein marker. 1: Intracellular protein of CSLH88-pCHKW strain (SDS-PAGE). 2: Extracellular protein of CSLH88-pCHKW strain (SDS-PAGE). 3: Intracellular protein of CSLH88-pCHKW strain (Western blot). 4: Intracellular protein of CSLH88-pCHKW strain (Western blot).

After the 24 h cultivation of the engineered strain CSLH88-pCHKW, intracellular and extracellular protein extractions were conducted, followed by SDS-PAGE and Western blot validation. The results obtained via SDS-PAGE demonstrate the presence of a significant amount of proteins in both the intracellular and extracellular fractions of the CSLH88-pCHKW strain, thus indicating the strain’s proficiency in normal protein secretion and expression. In addition, Western blotting revealed the presence of the target protein, *Cry3Aa-T*, in the extracellular and intracellular fractions of the CSLH88-pCHKW strain (**Figure 3**). These findings affirm the successful development of an engineered *Y. entomophaga* strain that can effectively secrete the insecticidal toxin protein *Cry3Aa-T*.

### 3.3 Genetic stability assessment of the engineered strain

To determine the genetic stability of the genetically engineered strain, CSLH88-pCHKW, successive subculturing was carried out, and the frequency of the *Cry3Aa-T* gene was subsequently determined. The data reveal that, in the absence of antibiotic selection pressure, the frequency of the CLSH88-pCHKW strain carrying the gene begins to decrease after 50 or more generations (**Table 1**).

**Table 1:** Detection of genetic stability of the strain.

Name of strain	Cultivation time (h)	Generation	Gene carrying rate (%)
CSLH88-pCHKW	12	8	100
	36	22	100
	60	36	100
	84	50	94

### 3.4 Analysis of the biological assay results of the engineered strain

**Figure 4:**
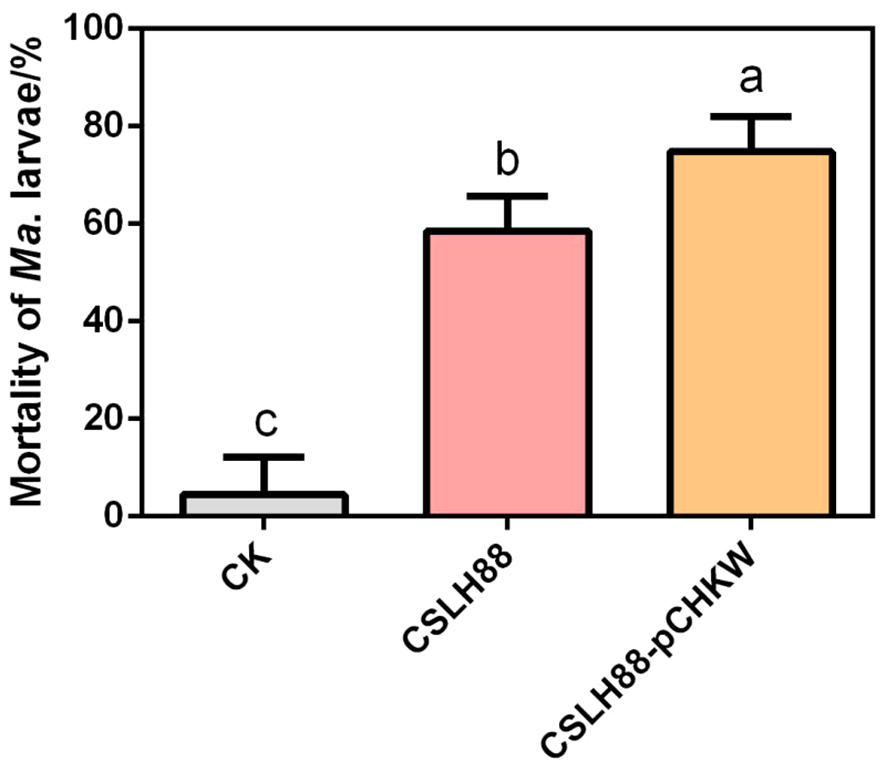
Mortality rate of *M. alternatus* larvae exposed to CSLH88 and CSLH88-pCHKW strains. The mortality rates of the larvae under different treatments with three biological replicates were observed and recorded (*p* < 0.05). Lowercase letters reflect a significant level of 5%.

The analysis of the biological assay results indicates that the unmodified *Y. entomophaga* strain CSLH88 exhibits some toxicity, as observed by an LC_50_ = 1.247×10^9^ CFU/mL. Conversely, the engineered strain CSLH88-pCHKW demonstrates a marked enhancement in toxicity, with an LC_50_ = 2.237×10^6^ CFU/mL (**Table 2**). Furthermore, we conducted a statistical analysis of the mortality rate of second-instar *M. alternatus* larvae fed with CSLH88 and CSLH88-pCHKW strains. Both strains significantly increased the mortality rate, with CSLH88-pCHKW exhibiting an average mortality rate of 75% (**Figure 4**).

**Table 2:** Insecticidal activity of the engineered bacteria against the larvae of *M. alternatus*.

Strain	LC50(CFU/mL)	95% confidence interval	Slope
CSLH88	1.247×10^9^	6.738×10^7^-3.474×10^11^	0.047
CSLH88-pCHKW	2.237×10^6^	1.156×10^5^-4.983×10^7^	0.04

### 3.5 Proliferation of CSLH88-pCHKW strain in the gut of *M. alternatus*

**Figure 5:**
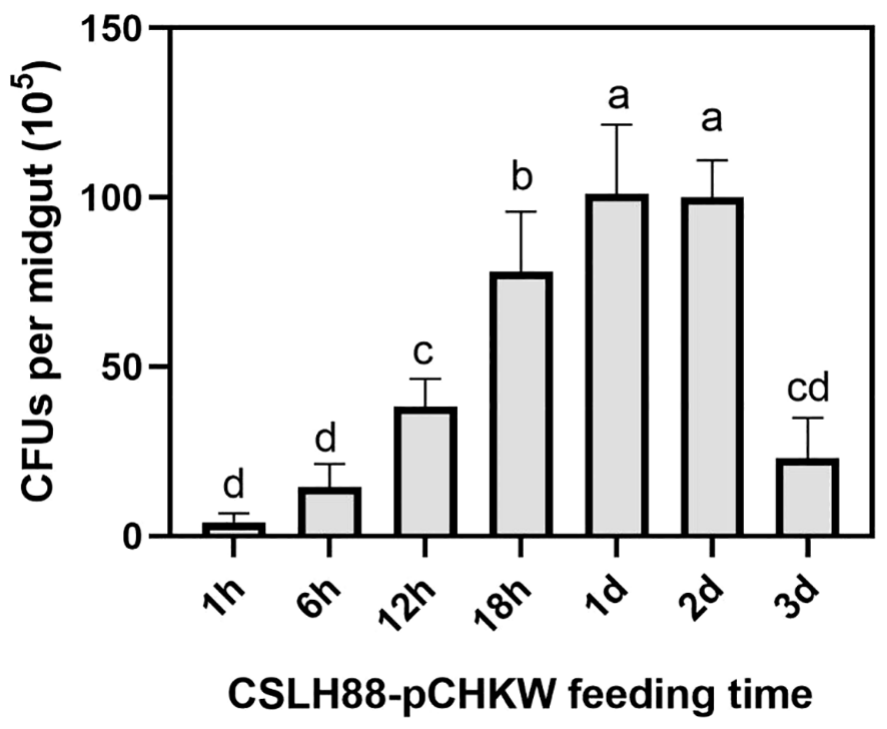
Proliferation of the CSLH88-pCHKW strain in the gut of *M. alternatus* larvae. The intestinal homogenates were spread on Kanamycin-containing agar plates to screen for colonies containing the toxin protein. Each experimental group was set with three biological replicates (*p* < 0.05). Lowercase letters reflect a significant level of 5%.

After feeding *M. alternatus* with the CSLH88-pCHKW strain, we periodically monitored the quantity of CSLH88-pCHKW in their intestines to determine if proliferation occurred. The grinding and plating of the intestines of the fed *M. alternatus* yielded results indicating a rapid proliferation of CSLH88-pCHKW within their intestines, with an increase of nearly 100-fold compared to the initial quantity (**Figure 5**).

## 4 Discussion

Bt is a bacterium widely utilized as an insect pathogen that produces two insecticidal toxins, Cyt and Cry, during the spore stage. Cry toxins have been extensively studied in the fields of biological pest control and genetically modified crops, showing remarkable insecticidal activity against diverse insect orders, including Lepidoptera (Amy and Estiati, 2020), Coleoptera (Rodríguez-González et al., 2020), Diptera (Qureshi et al., 2014), and nematodes (Guo et al., 2022). Furthermore, previous research has confirmed the high level of safety that Cry toxins exhibit in humans and mammals (Rubio-Infante and Moreno-Fierros, 2016). Lin et al. effectively synthesized CrylC and Cry2A toxins and successfully integrated them into rice plants. Consequently, a genetically modified variety of rice emerged, possessing resistance against Lepidopteran pests like *Chilo suppressalis* and *Scirpophaga incertulas* (Lin and Zhang, 2004a; Lin and Zhang, 2004b; Tang et al., 2006; Sujatha et al., 2009). In a separate study, Scott et al. incorporated the Cry51Aa2.834_16 toxin into cotton plants, showcasing its remarkable insecticidal activity against thrips and stinkbugs (Graham et al., 2019). Additionally, Khan et al. introduced the Cry14Ab toxin into soybeans, granting them resistance against the soybean cyst nematode (Kahn et al., 2021). Extensive research has been conducted on the insecticidal activity of Cry toxins; however, there is still a need for a highly potent toxin to effectively control *M. alternatus*, a significant pest of pine trees. Among Cry toxins, Cry3Aa demonstrates insecticidal activity against Coleopteran pests. Nevertheless, the Cry3Aa toxin has suboptimal insecticidal effects against *Apriona germari* and *Anoplophora glabripennis*, as indicated in recent studies (Chen et al., 2005; Guo et al., 2012). Our previous research found that the Cry3Aa toxin had limited toxicity against *M. alternatus* larvae. To enhance its potency, we made molecular modifications by considering the activation of digestive enzymes and the excessive enzymatic digestion occurring in the gut of *M. alternatus* larvae. As a result, we successfully developed a highly potent variant known as *Cry3Aa-T* toxin (Guo et al., 2020).

Symbiotic microorganisms in the gut are essential in regulating intestinal immunity and bodily functions and exerting diverse effects by producing microbial metabolites. Utilizing symbiotic bacteria to decrease vector capacity is a promising strategy (Ratcliffe et al., 2022). *Y. entomophaga*, a Gram-negative bacterium and an insect pathogen, exhibits inherent insecticidal activity and is pathogenic to various pests, including *Plutella xylostella* (Hurst et al., 2019). The pathogenicity of *Y. entomophaga* is attributed to the production of a complex called Yen-Tc (Hurst et al., 2011). Landsberg et al. found that a minimal Yen-Tc can be deadly for *P. xylostella* larvae (Landsberg et al., 2011). Previous research has shown that *Y. entomophaga* is effective against Coleopteran insects (Mansfield et al., 2020). The present experiment’s biological assay confirms that *Y. entomophaga* has pathogenic properties against *M. alternatus* larvae. The combination of this synergistic effect and the *Cry3Aa-T* toxin significantly enhances its efficacy. Its virulence has significantly increased compared to the original strain, and further screening of advantageous strains highly enriched in its intestines can be carried out as carrier strains to achieve better insecticidal effects.

This study aims to construct a secretion vector for the insecticidal toxin protein pCHKW by replacing the *GFP* gene in the expression vector pGHKW4 with the *Cry3Aa-T* gene. Utilizing our research group’s established toxin and excretion expression system, we successfully obtained the bacterium CSLH88-pCHKW via electroporation. The CSLH88-pCHKW strain exhibited the ability to excrete insecticidal toxins. We conducted stability testing through 50 generations to ensure the genetic stability of CSLH88-pCHKW. The results demonstrated that the CSLH88-pGHKW4 strain could consistently maintain stability. This recombinant plasmid stability is crucial for engineered bacteria’s functionality. A biological assay was conducted to evaluate the pathogenicity of the engineered strain compared to the unmodified CSLH88 strain, specifically for *M. alternatus* larvae. The results showed a notable increase in pathogenicity in the engineered strain. However, it should be noted that this biological assay took place in a controlled laboratory environment and may not wholly reflect the effectiveness of the modified strain in actual forest settings. Therefore, further experimental validation is required to verify and validate these findings. This study successfully developed an engineered strain of *Y. entomophaga* capable of producing and exhibiting the *Cry3Aa-T* toxin protein, which demonstrated high toxicity against *M. alternatus* larvae. Consequently, this engineered bacterium holds significant implications for further research efforts in *M. alternatus* control.

## 5 Conclusion

In this study, a genetically engineered strain CSLH88-pCHKW of the entomopathogenic bacterium was developed. SDS-PAGE and Western blot experiments confirmed that the strain successfully secretes the Cry3Aa-T insecticidal toxin protein. Passage experiments demonstrated stable genetic inheritance within the first 50 generations. Additionally, compared to the original entomopathogenic bacterium, the engineered strain showed significantly increased toxicity against *M. alternatus* larvae. In conclusion, study has constructed a highly toxic engineered bacterial strain against *M. alternatus* larvae, showing promising potential for biological control. This research provides a foundation and reference for further research and application of genetically engineered bacteria for pest control.

## Data availability statement

The original contributions presented in the study are included in the article/supplementary materials, further inquiries can be directed to the corresponding authors.

## Author contributions

XHa: Data curation, Methodology, Validation, Writing – original draft, Writing – review & editing. CH: Data curation, Methodology, Validation, Writing – original draft. HQ: Methodology, Validation, Writing – original draft. YZ: Methodology, Validation, Writing – original draft. XHu: Methodology, Validation, Writing – original draft. YW: Methodology, Validation, Writing – original draft. YL: Methodology, Validation, Writing – original draft. LX: Project administration, Writing – original draft. FZ: Funding acquisition, Resources, Writing – review & editing.
